# The “Pocket book of primary health care for children and adolescents”: WHO standards for improving paediatric and adolescent primary care in Europe, Central Asia and worldwide

**DOI:** 10.3389/fped.2024.1462303

**Published:** 2024-11-01

**Authors:** Carsten Krüger, Gottfried Huss, Farzana Yasmin, Ralf Weigel

**Affiliations:** ^1^ Friede Springer Endowed Professorship for Global Child Health, Department of Medicine, Faculty of Health, Witten/Herdecke University, Witten, Germany; ^2^ Past President of the European Confederation for Primary Care Paediatricians (ECPCP), Lyon, France

**Keywords:** adolescence, childhood, global child health, primary care, quality of care, standards

## Abstract

Paediatric primary care is more frequently provided both by physicians other than specialised paediatricians as well as non-physician cadres in Europe and globally. The quality of paediatric primary care including a focus on preventive measures is rather variable and often does not comply with agreed international standards. WHO Europe developed standards for paediatric primary care applicable to the WHO Europe region and globally to improve the provision and outcome of paediatric primary care across all settings. These standards were published in an open-access pocketbook and in an open-access app, both accessible free-of-charge. Although originally intended for use in the WHO Europe region, the pocketbook provides valuable information for all settings of paediatric primary care worldwide.

## Introduction

The provision of healthcare for newborns, children and adolescents is organized very differently around the world. The structure of so-called “primary” care in paediatrics and adolescent medicine, including a strong focus on prevention, varies considerably within the framework of diverse health systems (statal, parastatal, private/for-profit, not-for-profit, mixed forms). Particularly in countries of the Global South, this diverse group of patients is often treated in the outpatient sector by semi-skilled staff in primary health care, nursing staff or non-academically trained “medical” assistants (e.g., clinical officer, medical assistant, medical licentiate, assistant medical officer, assistant médical, técnicos de cirurgýa, feldshers).

**Figure 1:**
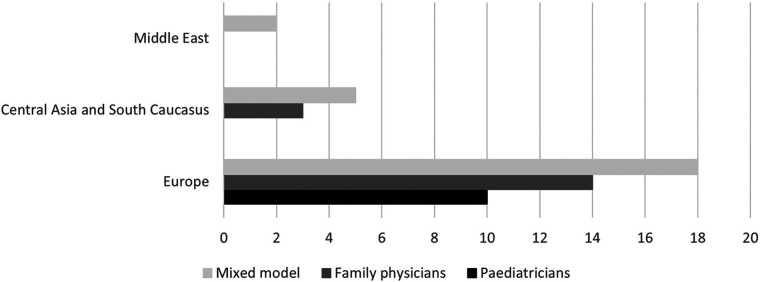
Primary paediatric care models in the 53 member states of the WHO Europe region (Montenegro: no information) (1).

In the WHO Europe region, which for geopolitical reasons comprises 53 countries in Europe, Central Asia, South Caucasus, and Middle East, there are large structural differences in primary paediatric care. In 19% of the countries paediatricians provide primary paediatric care, in 36% family physicians do so, and in the remaining 45% a mixed system is established (Table 1, Figure 1) (1).

**Table 1:** Member states of the WHO Europe region and their allocation to primary paediatric care models (1).

Europe		Central Asia South Caucasus	Middle East
Albania^b^	Malta^b^	Armenia^c^	Israel^c^
Andorra^b^	Moldova^c^	Azerbaijan^c^	Turkey^c^
Austria^c^	Monaco^c^	Georgia^c^	
Belarus^a^	Montenegro^d^	Kazakhstan^c^	
Belgium^c^	Netherlands^b^	Kyrgyzstan^b^	
Bosnia and Herzegowina^c^	North Macedonia^c^	Tajikistan^c^	
Bulgaria^c^	Norway^c^	Turkmenistan^b^	
Croatia^a^	Poland^c^	Uzbekistan^b^	
Cyprus^a^	Portugal^b^	(Russian Federation^a^)	
Czech Republic^a^	Romania^c^		
Denmark^b^	Russian Federation^a^		
Germany^a^	San Marino^c^		
Estonia^b^	Sweden^b^		
Finland^b^	Switzerland^c^		
France^c^	Serbia^c^		
Greece^a^	Slovakia^a^		
Hungary^c^	Slovenia^a^		
Ireland^b^	Spain^a^		
Island^b^	Ukraine^c^		
Italy^c^	United Kingdom of Great		
Latvia^b^	Britain and Northern		
Lithunia^c^	Ireland^b^		
Luxembourg^b^			

^a^ paediatricians.

^b^ family physicians.

^c^ mixed system.

^d^ no information.

## Policy options/actionable recommendations

### Challenges

Children and adolescents often receive suboptimal healthcare in several countries of the WHO Europe region (2–6). The reasons are manifold. The paediatric training for general primary care providers is usually too short. The Integrated Management of Childhood Illness (IMCI) strategy has almost nowhere been fully implemented limiting its impact on outpatient care (3). Outpatient facilities are frequently overcrowded which was particularly true during the COVID-19 pandemic. Treatment is often not evidence-based or in line with guidelines. In addition to poly-pharmacotherapy (2), several countries suffer from resource-related constraints and insufficient immunisation coverage. Preventive measures, e.g., to improve vaccination coverage or to mitigate obesity and overweight, are not well developed (7). Many children are unnecessarily hospitalised, partly because of lack of reliable diagnostic facilities or fear of deterioration after sending them home in the outpatient setting, subjecting them to inadequate treatment and increasing the burden on the health system and the families (2, 3). Numerous studies from the WHO Europe region show that the allocation of resources, diagnostics and medication, particularly antibiotic therapy, are used too often indiscriminately, with all the known consequences (2–6). All these factors contribute to limiting the pathway to a healthy life and development of the child's full potential (8).

### The response of WHO Europe

**Figure 2:**
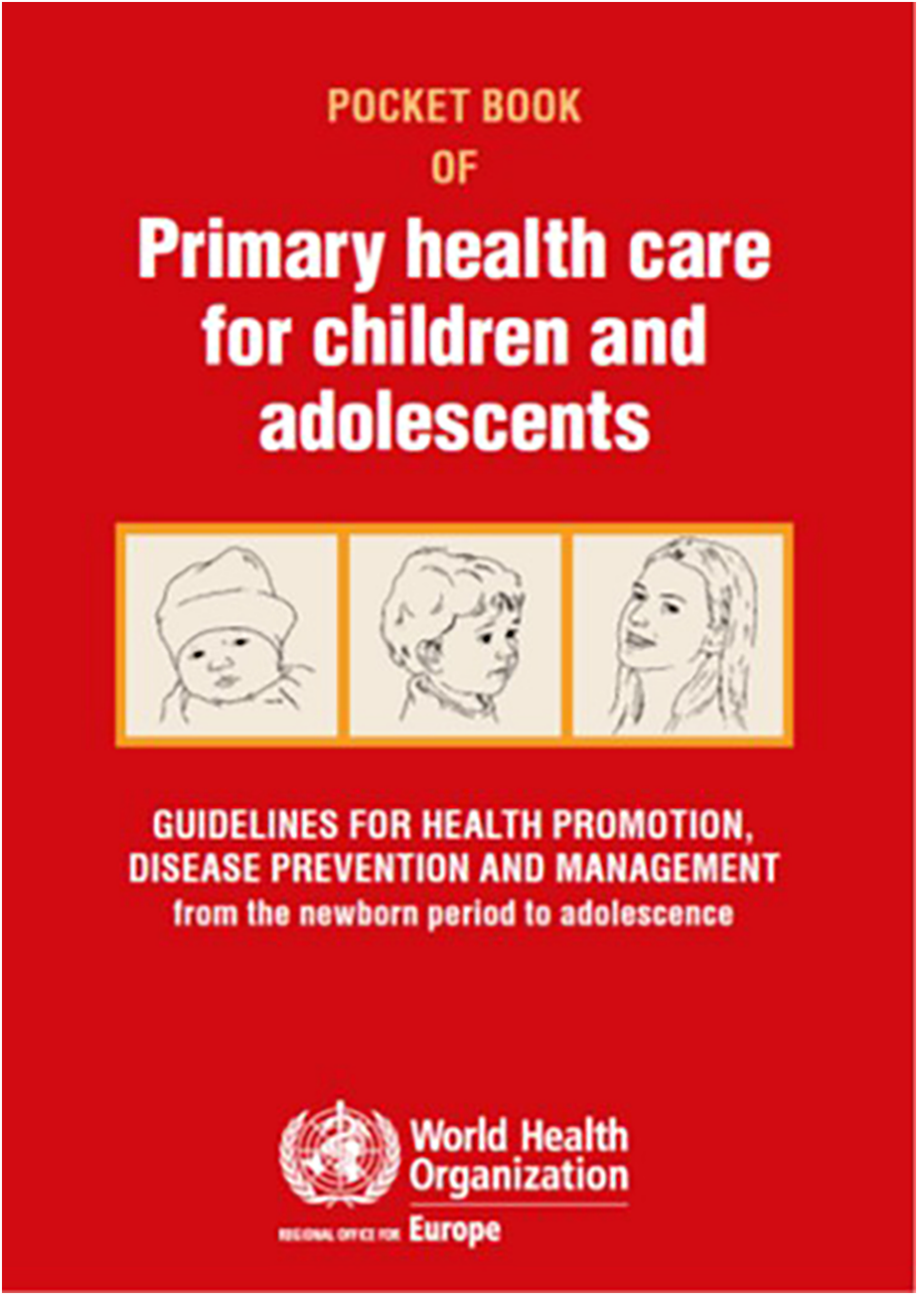
“Pocket Book of Primary Health Care for Children and Adolescents”.

To improve this situation and to enable the best possible, evidence-based standardisation of prevention, diagnosis and treatment from the neonatal period to adolescence that is largely independent of the health care provider's qualification level, WHO Europe published the “Pocket Book of Primary Health Care for Children and Adolescents” in 2022 (Figure 2) (9). It comprises nine chapters and ten appendices on approximately 950 pages and is available online (Table 2) (9). Conceptually, the book is based on the established “WHO Pocket Book of Hospital Care for Children” (10), which provides structured, evidence-based treatment guidelines for common and/or potentially life-threatening diseases in the neonatal period and childhood, particularly for health systems of countries in the Global South. In terms of concept, both publications address not only physicians but also other professionals, such as nursing staff and “medical” assistants.

**Table 2:** “‘Pocket book of primary health care for children and adolescents”: chapters and appendices.

1. Providing Care from Birth through Adolescence
2. Diagnostic Approaches
3. Well Child Visits
4. Health Promotion and Disease Prevention from Birth through Adolescence
5. Newborn Health
6. The Child or Adolescent presenting with a Specific Complaint or Symptom
7. Diseases and Conditions
8. Adolescent Health
9. Emergencies and Trauma
Annexes
1. Organization of care level
2. Practical procedures
3. WHO growth charts
4. Drug dosages
5. Comparison of systemic corticosteroids
6. Oral rehydration solution (ORS)
7. Intravenous fluids
8. Asthma action plan
9. Hypersensitivity reactions
10. Equipment sizes for young children

Paediatric and non-paediatric experts and professional associations from Europe were involved in the development and editing under the leadership of the Child and Adolescent Health Department at WHO Europe, Copenhagen. All recommendations were reviewed and updated by renowned experts. Finally, the text had to undergo an external and internal revision process according to WHO standards and following the GRADE approach to ensure that all statements and recommendations are up to date and of high quality (11).

The individual chapters are organised according to a uniform standard, as far as possible for the topic: a brief definition is followed by information on the medical history, clinical picture, necessary examinations/diagnostics, differential diagnosis, and treatment. In addition, there is often information on how to proceed after the initial treatment, how to arrange follow-up and on long-term care for chronic diseases. The information on diagnosis and treatment is particularly important, as this is intended to counteract over- or under-treatment.

In line with the 45-year-old WHO concept of “Primary Health Care” (12), the book places particularly emphasis on health education and prevention, but at the same time provides detailed instructions for high-quality paediatric acute care. The German paediatric standard was used as a blueprint for the content and frequency of preventive well-child visits. To substantiate the evidence of preventive measures in the neonatal period, childhood and adolescence, authors from WHO Europe and the Global Child Health Working Group at the Witten/Herdecke University (www.uni-wh.de/gch), with the support of the European Confederation of Primary Care Paediatricians (www. ecpcp.eu), analysed and published the current state of the art on ten selected topics (prevention with vitamin K, vitamin D, fluoride; prevention of SIDS, accidents; screening for congenital heart defects, anaemia, visual disorders, speech/hearing disorders and autism spectrum disorders) (13).

Considering the overpopulation of (not only) the paediatric emergency departments, especially during the COVID-19 pandemic years, the pocketbook is equally strong on detailed guidance for acute (emergency) care and interventions in the paediatric primary care setting. Strengthening evidence-based treatment in childhood and adolescence is the declared aim of the pocketbook and is intended to help remedy the overuse, underuse and misuse of drugs, particularly antibiotics, that can still be observed in the WHO Europe region (2–6, 8). Although not explicitly mentioned, modern telemedicine applications will have to play an increasingly important part in the provision of paediatric primary care in the 21st century.

Obviously, there are difficulties in implementing and applying the content in the 53 countries of the WHO Europe region. So far, the book is only available in English, Ukrainian and Russian; translations into the languages of the member states are therefore necessary for widespread dissemination and application. The content will have to be adapted to the respective national healthcare systems. Different professionals, some outside the field of medicine who provide “primary” medical care to children and adolescents within the WHO Europe region, need to be considered, too. National guidelines, drug supply regulations and sector structures also need to be considered. Nevertheless, the respective healthcare system can orientate itself on the guidelines and address deficits in the respective country accordingly.

## Conclusion: scope for a wider audience

Although initially intended for the WHO Europe region including high-income countries, the book provides a good basis for ensuring improved paediatric care worldwide, particularly in the outpatient setting, thereby expanding on the already existing IMCI approach. This is even more true as in most countries of the Global South, children and adolescents are rarely treated by specialised paediatric medical staff in primary care.

In the busy daily routine, the individual chapters can be quickly consulted whether one's own assessment and dosage of medication is correct or whether one should consider other differential diagnoses. General practitioners involved in the care of children and adolescents can benefit from the comprehensive but short compilation of all aspects of prevention and treatment of common outpatient conditions.

The pocketbook differs significantly from other heavyweight and detailed clinical paediatric textbooks. It covers prevention like well-child visits and vaccination in detail and focuses on the symptom-oriented management of common consultation problems in a practical way. At the end of April 2024, WHO released an app for the pocketbook, which provides free access to its content for everyone and is a significant step forward for a wider distribution of its content, aligned to the current information strategies in the healthcare sector. The app is available free of charge in the relevant app stores (9).

For doctors and nurses planning to work in humanitarian aid or medical development cooperation, this compact pocketbook and its app are definitely worth having in their hand luggage. However, there are no chapters like on malaria, dengue or other specific tropical diseases. Their omission is based on the geographical location of the WHO Europe member states, but it does not detract from the great value of the pocketbook. What is common in Europe is also common in the countries of the Global South. The primary care provider for children and adolescents in every country in the world can draw on a rich, condensed pool of up-to-date information in this handbook.
